# Diet-related inflammation increases the odds of multiple sclerosis: Results from a large population-based prevalent case-control study in Jordan

**DOI:** 10.3389/fnut.2023.1098883

**Published:** 2023-04-05

**Authors:** Omar A. Alhaj, Khaled Trabelsi, Abdallah M. Younes, Nitin Shivappa, Nicola L. Bragazzi, James R. Hebert, Haitham A. Jahrami

**Affiliations:** ^1^Department of Nutrition, Faculty of Pharmacy and Medical Sciences, University of Petra, Amman, Jordan; ^2^High Institute of Sport and Physical Education of Sfax, University of Sfax, Sfax, Tunisia; ^3^Consultant Neurologist, Amman, Jordan; ^4^Department of Epidemiology and Biostatistics, Arnold School of Public Health, University of South Carolina, Columbia, SC, United States; ^5^The Cancer Prevention and Control Program, University of South Carolina, Columbia, SC, United States; ^6^Department of Nutrition, Connecting Health Innovations LLC, Columbia, SC, United States; ^7^Laboratory for Industrial and Applied Mathematics, Department of Statistics, York University, Toronto, ON, Canada; ^8^Human Nutrition Unit, Department of Food and Drugs, University of Parma Medical School, Parma, Italy; ^9^Department of Psychiatry, College of Medicine and Medical Sciences, Arabian Gulf University, Manama, Bahrain; ^10^Ministry of Health, Government Hospitals, Manama, Bahrain

**Keywords:** dietary inflammatory index (DII), nutrition, multiple sclerosis, macronutrients, micronutrients

## Abstract

**Background:**

Multiple sclerosis, a chronic inflammatory disease in young and middle-aged adults, is one of the leading causes of non-traumatic disability in adults. Diet is known to have an important role in the modulating inflammatory processes and influencing molecular pathways.

**Purpose:**

This study aims to examine the association of the inflammatory capacity of diet measured by DII with MS in Jordan.

**Methods:**

This prevalent case-control study included participants of both sexes, aged between 20 and 60 years. The cases (*n* = 541) had a confirmed diagnosis of prevalent Multiple Sclerosis (MS) in the previous 3 years, and controls (*n* = 607) were apparently healthy individuals matched on sex and age (42 ± 4 years). A validated Arabic food frequency questionnaire (FFQ) was utilized to obtain estimated dietary intake. Dietary data from the FFQ were analyzed using ESHA’s Food Processor^®^ nutrition analysis software, and the results were used to calculate the DII scores. Logistic regression analyses, controlling for covariates such as age, sex, body mass index, and smoking status, were used to measure the association between DII score and MS outcomes.

**Results:**

Cases represent a mixed sample of MS phenotypes and controls were comparable on age and sex. However, controls tended to be taller, lighter, had a lower BMI, and had a lower smoking rate. After controlling for age, BMI, sex, and smoking status, there was a consistent increase in MS risk according to DII score, with a 10-fold increase in odds in quartile 4 vs. quartile 1 [OR_quartile 4vs1_ = 10.17 (95% CI: 6.88; 15.04)]. For each point increase in DII score, there was nearly a doubling of odds [OR_1_ = 1.75 (95% CI: 1.59; 1.92)]. Individual nutrients and food values aligned according to their contribution to the DII score calculations.

**Conclusion:**

The findings of this study, obtained in MS patients with varied illness duration over the previous 3 years, are consistent with an association between the overall inflammatory potential of diet and MS odds. Our findings among MS participants showed a significantly more pro-inflammatory DII scores than age- and sex-matched controls. Our results also suggest that MS group had a diet rich in pro-inflammatory foods and nutrients.

## 1. Introduction

Multiple sclerosis (MS), a neurodegenerative inflammatory disease, is one of the leading causes of non-traumatic disability in young adults (1). According to the latest edition of MS Atlas, the prevalence of MS worldwide in 2020 is 2.8 million (2). According to a recent study, 38.4% of Jordanian society are currently treated for several illnesses including immune-mediated diseases (3), the prevalence of MS disease in the capital city of Amman/Jordan was 0.039% (4). As a country with a medium-high MS risk (4), Jordan provides an ideal setting to test whether there is an association between MS and food consumption and nutrition awareness (5). Various non-modifiable variables, including female sex and genetic factors, have been linked to the development of MS. Still, the focus on modifiable environmental risk factors represent an important practical way to address the prevention of MS (6). Vitamin D deficiency (7), smoking (8), and high body mass index (BMI) (9) have all been identified as potentially important. Recently, Epstein-Barr virus also was reported as a putative major risk factor for MS (10, 11). Diet appears to be a possible co-factor in the inflammatory process, influencing molecular pathways and gut microbiota (12). A healthy diet and supplement use have been found to reduce pro-inflammatory cytokine production and, as a result, reduce inflammation (13), fatigue, body mass index (BMI), low-density lipoprotein cholesterol, total cholesterol, and insulin level (14) in people with MS. Moreover, adherence to the Mediterranean diet was found to have a beneficial effect on MS course and disability (15). The DII was developed to quantify the effect of diet on inflammation (16). Several food parameters exhibited anti-inflammatory DII including fiber, Mg, niacin, flavonoids, (16). In another case-control study, a lower incidence of primary progressive MS was linked to increased consumption of anti-inflammatory (low-DII) foods including dairy, seafood, vegetables, fruits, poultry, and nuts as well as calcium, iron, folic acid, vitamin B12, and vitamin C supplements (17). While, pro-inflammatory food parameters include carbohydrate, cholesterol, total fat, saturated fat, trans fat (16), and red meat (18). Abdollahpour et al. (19) reported that an increased risk of MS was linked to a pro-inflammatory diet throughout adolescence, as measured by higher DII scores. Similar results also were reported by Shivappa et al. (20), who reported that MS odds are directly correlated to high DII. In another study, the nutritional intake of MS patients was found to produce higher rates of diet-associated inflammation, as indicated by higher DII scores, consequently contributing to the disability level (21).

Studies investigating the associations between diet and intakes of a particular nutrient or their plasma concentrations and MS, though potentially important, are very few in number. The National Multiple Sclerosis Society published a guideline for vitamins and minerals in 2011 that can be effective when dealing with MS patients (22). According to a systematic review by Torkildsen et al. (23), vitamin E and vitamin A intake were shown to be important in the pathogenesis of MS; the benefit of vitamin A in managing MS is supported by another published review (24).

Vitamins B2 and B6 in adequate intakes were found to prevent MS disease (25); folate and vitamin B12 also were proposed as protective factors (26). On the other hand, vitamin B2 supplementation in another randomized clinical trial did not reduce the severity of disability status (27). Restriction of saturated fat also has been shown to have a beneficial effect in MS patients and therefore has been evaluated in several studies; however, results remain inconclusive (28). This study aims to examine the association of the inflammatory capacity of diet measured by DII with MS in Jordan.

## 2. Materials and methods

### 2.1. Study design, participants, and setting

This prevalent case-control study was designed and reported using Strengthening the Reporting of Observational Studies in Epidemiology—Nutritional Epidemiology (STROBE-nut) guideline, which refers to a simple reporting guideline checklist used to improve the quality of the research design (29). This study included 541 patients diagnosed with MS by a multidisciplinary neurology team in Jordan using the International Classification of Diseases version 10 (ICD-10) and following the revised McDonald criteria in 2017 for MS Diagnosis (30). Prior to recruitment of healthy controls, the clinical signs and characteristics of MS and other severe medical comorbidities were clarified to the participants. This verification was done to avoid misidentifying and misclassifying of healthy participants as cases participants (19). Healthy controls (not from MS family) without self-report medical records or symptoms of MS (*n* = 607) matched on sex and age (average 42 years) were recruited from the general population from different areas of Amman. Simple random sampling was used to select the MS cases from the study site’s case registry located in Amman, Jordan. The recruitment process took place in the out-patient department, between the months of March and June in the year 2021. Data collected reflect a duration of illness that varied from 1 to 3 years.

Outpatients of both sexes diagnosed with MS, aged between 20 and 60 years, able to provide consent, and express willingness to participate in the study were eligible to participate in the study. Those patients having severe medical comorbidities (cancer, motor neuron diseases, chronic kidney disease, psychiatric disorders), pregnant women, and those participating in other experimental protocols were excluded.

### 2.2. Study size

Based on previous similar research (8), we estimated that a sample size of 100 patients would be required to answer the research hypothesis for our prevalent case-control analysis. Patients with MS are two times (odds ratio = 2.0) more likely to be on the extreme end of the pro-inflammatory diet (20). Therefore, the sample size calculator included the following assumptions α = 5%, β = 20% to achieve 80% power. We decided to include about 500 patients per group to increase the power further.

The Research Ethics Committee, University of Petra, Jordan (UOP/REC: Q1R/11/2020) granted ethical approval for the study. Before any data were collected, all participants signed a written informed consent form. The study adhered with the Declaration of Helsinki ethical principles for medical research involving human subjects.

### 2.3. Study tools and measurements

The following assessments were undertaken during the clinical interview: demographics, and anthropometrics measurements (weight and height) using Seca digital stadiometer, which measures body weight with 0.1 Kg accuracy and height to the nearest ± 0.1 cm (31). Body mass index (BMI) was calculated based on measured weight and height by dividing body weight in kg by the squared height in meters. The BMI of subjects were then classified as underweight (BMI < 18.5 kg/m^2^), normal (BMI < 24.9 kg/m^2^; BMI ≥ 25.0), overweight (25), or obese <29.9; BMI ≥ 30.0, respectively by the World Health Organization (32).

The participants received one study questionnaire survey that consisted of the detailed dietary habits and other lifestyle aspects. The dietary intake was assessed using a validated Arabic food frequency questionnaire (FFQ) with Cronbach alpha and McDonald omega of >0.9 (33, 34). The FFQ is described in detail in another publication (33–35). Briefly, it includes thirty food items and eight beverages, the FFQ question asked about the average frequency of a certain food item throughout the past month as well as standard serving. The collected responses of the consumed frequency of a specific serving size was standardized using food images to assess a standard unit for portions.

Dummy models were utilized to determine a standard unit for portions to standardize data collection further. The FFQ, which measured food intake over the previous 4 weeks, was divided into five major food groups, which were further divided into 32 categories. According to a recent systematic review and meta-analysis, using FFQs (typically from the previous month) is a reliable method in nutritional epidemiological studies (36).

ESHA’s Food Processor^®^ nutrition analysis software was utilized to analyze dietary of the FFQ, and Dietary Inflammatory Index (DII^®^) scores were calculated from these data. A total of 32 food parameters, of the possible 45, were used to calculate the DII score. These are: Energy (kcal); Protein (g); Carbohydrate (g); Fiber (g); Cholesterol (mg); Total Fat (g); Saturated Fat (g); Monounsaturated Fat (g); Polyunsaturated Fat (g); Calcium (mg); Iron (mg); Magnesium (mg); Sodium (mg); Phosphorous (mg); Potassium (mg); Zinc (mg); Niacin B3 (mg); Thiamin B1 (mg); Riboflavin B2 (mg); Vitamin A (RE); Pyridoxine B6 (mg); Cobalamin B12 (μg); Vitamin C (mg); Omega-3 (g); Omega-6 (g); Vitamin D (IU); Vitamin E (mg); Folic acid (μg); Selenium (μg); Caffeine (mg); β-carotene (μg); Alcohol (g).

An in-depth description of the DII (37) and the energy-adjusted DII (E-DII™) can be found elsewhere (38). Briefly, the DII scoring algorithm was based on a careful review of the literature through which 1943 articles identified 45 food parameters (i.e., macronutrients including specific categories of fatty acids, carbohydrate, and proteins; macronutrients including vitamins and minerals; flavonoids; and whole food items including herbs and spices) as having sufficiently robust literature in relation to six inflammatory biomarkers—i.e., interleukins (IL)-1β, –4, –6, –10, tumor-necrosis factor-alpha (TNFα), and C-reactive protein (CRP). As noted, self-report values for 32 of these food parameters were available from the FFQ. These were translated into z-scores using a global comparative database consisting of data from 11 countries by subtracting from the individual’s self-report value the mean of the global database then dividing by the standard deviation. These scores were then converted to proportions and centered on zero by doubling each and subtracting one. These centered proportions were then multiplied by their respective coefficients (overall food parameter-specific inflammatory effect scores) to obtain DII scores for each food parameter. These were summed to obtain the overall DII score. E-DII scores were calculated using the density approach by calculating DII per 1,000 kcal consumption. This employed the same procedure for scoring, but relies on an energy-adjusted global comparison database (38). These DII and E-DII scores have a potential range from approximately –9 to + 8; i.e., from minimally to maximally pro-inflammatory, respectively. The DII and E-DII are scored similarly and scaled identically; so, the scores are comparable across studies. For the E-DII, energy was in the denominator; so, 31 parameters were used for computation. The decision to use the DII was based on model goodness of fit/explanatory ability.

### 2.4. Statistical methods

Before beginning the analyses, Shapiro–Wilk test was utilized to visualize the data for tested normality and formally. Means and standard deviations are reported for continuous variables, and counts and percentages are reported for categorical variables. The independent sample *t*-test was used for continuous variables and the Chi-square test was used for categorical variables to compare the two independent groups. With age, sex, body mass index, and smoking status as covariates, multinomial logistic regression analysis was performed on DII as the outcome variable and group assignments of the participants according to DII quartiles as the predictor. All data analysis was done using STATA 17.0 software and, as noted, statistical significance was set at α = 0.05, two-sided. We used three measures of model fit for regression analyses to determine if our data better fit DII or E-DII. The fit indices included R-squared (R^2^), Akaike Information Criterion (AIC); and Bayesian Information Criterion (BIC) (39). To explain further, when performing regression analysis, it is important to assess which model best fits the data. To determine if the data better fits the DII or E-DII model, three different measures of model fit must be used. R^2^ gives the proportion of variation in the response variable which is explained by the model. The higher the R^2^, the better the model fits the data. By taking into account these three measures of model fit, we can reasonably assess whether the data fits the DII or E-DII model better. The results provide a better understanding of the data and help inform further analysis. This can help to ensure that the best fitting model is utilized and the results are as accurate as possible.

## 3. Results

### 3.1. Descriptive data

Descriptive data for both cases and controls are shown in Table 1. MS is a more common disease in females compared to males as is reflected in the lower percentage of males among MS cases and sex-matched controls. There also was no significant difference between the cases and controls in terms of age, the other variable on which we matched. However, large and statistically significant differences were observed in the anthropometric measurements. Cases were more likely to be obese compared to controls (21.07 vs. 10.21%). Cases also were more likely to be current smokers compared to controls (24.95 vs. 16.31%). Relapsing remitting multiple sclerosis (RRMS) represents most (≈85%) of the studied cases, while the prevalence of primary progressive multiple sclerosis (PPMS) and secondary progressive multiple sclerosis (SPMS) was 15%.

**TABLE 1:** Anthropometric and sociodemographic characteristics of cases with multiple sclerosis (MS) and their counterpart healthy controls, Jordan, March to June, 2021.

Variable*	Cases *n* = 541	Controls *n* = 607	*P*-value**	Effect size***
Age (years)	41.82 ± 3.98	42.1 ± 4.1	0.58	0.03
BMI (kg/m^2^)	26.07 ± 5.42	24.64 ± 4.36	<0.001	–1.29
Male	171 (31.61%)	191 (31.47%)	0.96	0.001
Obese	114 (21.07%)	62 (10.21%)	<0.001	0.16
Smoking	135 (24.95%)	99 (16.31%)	<0.001	0.10
	**MS subtypes**	**Proportion**	**Mean**	**SD**
DII	RRMS	84.47%	0.69	1.50
	PPMS/SPMS	15.53%	0.78	1.63
E-DII	RRMS	84.47%	–0.89	0.75
	PPMS/SPMS	15.53%	–0.89	0.75

*Frequency count and (%) OR Mean ± SD. **Independent samples *t*-test or Pearson’s Chi^2^, significant at *p* < 0.05. ***Cohen’s d or Cramer’s V. BMI, body mass index.

### 3.2. Main results

The results in Table 2 are in line with the predicted direction between cases and control participants, except for the lack of effect of carbohydrate, fiber, and selenium. The DII scores are shown in Table 2. The decision to use the DII instead of the E-DII score was based on model goodness of fit using the AIC = 1,398, BIC = 1,427, R2 = 0.22 for DII and AIC = 1,499, BIC = 1,528, R2 = 0.11 for E-DII. The ORs and 95% CIs for MS status by quartiles of DII are shown in Table 3. Participants in the most pro-inflammatory group (Quartile 4) were about ten times more likely to have MS than participants in the most anti-inflammatory group (Quartile 1) [OR 10.17 (95% CI 6.88; 15.04); *P*-trend = 0.001] when DII was expressed as a continuous variable.

**TABLE 2:** Distribution of dietary factors, nutrients, dietary inflammatory index (DII), energy dietary inflammatory index (E-DII) for cases and control subjects.

Nutrient or dietary factor*	Cases *n* = 541	Controls *n* = 607	*P*-value**	Effect size***
Energy (kcal)	1257 ± 392.82	1813.25 ± 459.97	<0.001	–0.35
Protein (g)	65.89 ± 26.53	74.8 ± 24.61	<0.001	–1.75
Carbohydrate (g)	147.97 ± 54.76	258.42 ± 69.77	0.72	0.02
Fiber (g)	16.63 ± 6.93	16.47 ± 7.94	0.27	–0.07
Cholesterol (mg)	189.29 ± 91.3	195.22 ± 88.66	<0.001	–0.46
Total Fat (g)	44.62 ± 16.01	53.38 ± 21.32	<0.001	–0.46
Saturated Fat (g)	18.72 ± 6.97	22.27 ± 8.32	<0.001	–0.31
Monounsaturated Fat (g)	14.88 ± 5.99	16.87 ± 6.94	<0.001	–0.48
Polyunsaturated Fat (g)	11.02 ± 4.52	14.24 ± 8.12	<0.001	–0.67
Calcium (mg)	306.08 ± 122.75	409.31 ± 178.47	<0.001	–0.21
Iron (mg)	24.35 ± 10.35	26.64 ± 11.67	<0.001	–0.44
Magnesium (mg)	177.19 ± 59.56	204.2 ± 64.18	<0.001	–0.28
Sodium (mg)	1287.6 ± 510.6	1443.48 ± 590.97	<0.001	–1.00
Phosphorous (mg)	480.07 ± 182.41	664.71 ± 185.19	<0.001	–0.62
Potassium (mg)	1867.3 ± 538.96	2240.06 ± 652.98	<0.001	–0.44
Zinc (mg)	5.79 ± 2.76	6.95 ± 2.51	<0.001	–0.82
Niacin B3 (mg)	13.34 ± 6	18.4 ± 6.38	<0.001	–1.65
Thiamin B1 (mg)	0.69 ± 0.32	1.29 ± 0.4	<0.001	–1.00
Riboflavin B2 (mg)	0.78 ± 0.32	1.13 ± 0.38	<0.001	–1.03
Vitamin A (RE)	493.17 ± 300.61	829.14 ± 345.83	<0.001	–0.74
Pyridoxine B6 (mg)	1.39 ± 0.5	1.76 ± 0.49	<0.001	–0.70
Cobalamin B12 (mcg)	3.19 ± 0.69	3.67 ± 0.68	<0.001	–0.75
Vitamin C (mg)	28.94 ± 14.39	42.19 ± 19.97	<0.001	–0.46
Omega-3 (g)	1.97 ± 0.65	2.28 ± 0.69	<0.001	–0.48
Omega-6 (g)	20.31 ± 4.05	22.58 ± 5.17	<0.001	–0.37
Vitamin D (IU)	231.54 ± 91.18	263.76 ± 84.87	<0.001	–0.98
Vitamin E (mg)	13.30 ± 13.37	27.77 ± 16	<0.001	–1.03
Folic acid (mcg)	43.27 ± 38.07	84.4 ± 41.43	<0.001	–0.37
Selenium (mcg)	114.02 ± 44.56	129.92 ± 41.53	0.08	–0.10
Caffeine (mg)	58.23 ± 64.21	64.64 ± 58.82	<0.001	–1.03
β-carotene (mcg)	6195.24 ± 3318.67	9751.46 ± 3547.94	<0.001	–0.29
Alcohol (g)	0.29 ± 1.36	1.14 ± 3.78	<0.001	0.80
DII score	0.70 ± 1.52	–0.39 ± 1.21	<0.001	–0.47
E-DII score	–0.89 ± 0.75	–0.55 ± 0.74	<0.001	–0.35

*Mean ± SD. **Independent samples *t*-test, significant at *p* < 0.05. ***Cohen’s d, DII, dietary inflammatory index, E-DII, energy dietary inflammatory index.

**TABLE 3:** Odds ratios (OR) and confidence intervals for quartiles of dietary inflammatory index (DII) (and DII as continuous) associated with the diet of patients with multiple sclerosis.

	Quartile 1	Quartile 2	Quartile 3	Quartile 4	DII (continuous)
DII range	≤-0.98	–0.97; –0.15	–0.14; 1.43	≥1.44	–
Cases/controls	80/208	120/218	112/122	229/59	–
Multivariate*	1 (ref)	1.44 (1.02; 2.04	2.49 (1.72; 3.61	10.17 (6.88; 15.04)	1.75 (1.60; 1.92)

*P*-trend = 0.001. *Adjusted for covariates: age, BMI, body mass index, sex, smoking status, DII, dietary inflammatory index.

## 4. Discussion

The prevalence of RRMS, PPMS, and SPMS in our study agree with the local trend reported in Jordan (4). Our results suggest that the FFQ-derived DII scores were associated with higher likelihood of MS disease. These results agree with those from other MS studies (19). Dietary indices and dietary pattern analysis have been proposed as alternative and complementary approaches to examine the association between diet and chronic diseases (40, 41). Compared to specific foods or nutrients, dietary indices or patterns theoretically provide a more comprehensive picture of food and nutrient consumption and may therefore be a promising approach of predicting disease risk (42, 43). Furthermore, changing the diet could have promising results on MS disease; for example adherence to a modified Mediterranean diet for 6 months has been shown to improve the dietary inflammatory status and fatigue severity in relapsing-remitting MS patients (44). On the other hand, the traditional Iranian diet has no positive impact on dietary inflammation and modified fatigue impact scale score (44). A cross-sectional research study conducted in Brazil on 137 MS cases revealed that DII was unable to change the clinical course of the disease (21). In the current study, participants with MS had a significantly more pro-inflammatory DII score compared to controls who were age- and sex-matched; i.e., DII scores of 0.70 ± 1.52 and –0.39 ± 1.21, respectively. Our results might be generalized to patients with varied illness duration over the previous 3 years. Participants with MS had a significantly more pro-inflammatory DII than age- and sex-matched controls. In two recent studies, a higher risk of demyelinating autoimmune diseases, including MS, were linked to eating a pro-inflammatory diet (45, 46). In our study, findings among participants with MS confirmed these results; i.e., they had a significantly more pro-inflammatory DII scores than age- and sex-matched controls. Our results suggest that MS group had a diet rich in pro-inflammatory foods and nutrients, which would significantly have increased IL-1β, IL-6, TNF-α, or CRP, or decreased IL-4 or IL-10 (16). Diets of healthy individuals appear to be less inflammatory than those of MS patients (21). Riccio and Rossano (47) proposed two potential mechanisms to explain the correlation between the inflammatory process due to a high DII and odds of MS. First, pro-inflammatory foods that are typical of an energy-dense, high-saturated-fat, and anabolism-promoting diet are metabolized in a direct biological pathway to inflammation (47). The second is an indirect effect brought on by the microbiota of the gut. In this case, a consistently energy-dense diet deficient in complex carbohydrates threatens the balance of the gut microbiota, resulting in a dysbiotic, inflammatory gut and, ultimately leads to systemic inflammation (47).

The DII was developed specifically to quantify the inflammatory effect of food on inflammation. In over 900 studies it has shown to have a good predictive ability with regard to conditions that are known or thought to be related to inflammation (48–51). Similar to our results, an earlier study that validated the DII in a total sample of 494 adults of both sexes using dietary data from 24 h dietary recalls and high-sensitivity C-reactive protein (hs-CRP) as a marker of inflammation discovered a correlation between the DII and hs-CRP (52). The majority of previously published dietary studies in MS focused on single foods or nutrients (19). Our findings are in line with the effects of individual food parameters (such as micronutrients, macronutrients, and other dietary components) on overall diet-related inflammation. The DII is superior in that it incorporates the most commonly consumed bioactive components including flavonoids, spices and tea, in addition to essential micronutrients and macronutrients (16). Abdollahpour et al. reported the potential Association between DII and MS onset (19); subjects with a diet high in inflammatory foods are more likely to experience MS onset than those with more anti-inflammatory foods. In the current study, MS participants consumed lower amounts of monounsaturated and polyunsaturated fats compared to the control group and even lower than recommended Dietary Reference Intakes (DRI). Both fats (monounsaturated and polyunsaturated) were previously reported to exhibit anti-inflammatory potential and provide a protective role for MS development (53, 54). Similarly, lower intakes of fat-soluble vitamins (D, A, and E) was noticed in MS participants which is lower than recommended Dietary Reference Intakes (DRI), which could increase the severity and symptoms of MS disease. All fat-soluble vitamins were effective in various animal studies of MS, and vitamins A and E have shown to provide biological properties that may be important to MS pathogenesis (23). According to a review of recent publications, vitamin A has strong evidence (33 studies) of suppressing immune responses and improving remyelination in MS patients (55). In another recent systematic review, researchers recommended MS patients to follow plant-based diets and limits animal foods, fried and processed foods (56).

### Strengths and limitations

The current results from a large population-based prevalent case-control study in Jordan add to the existing MS literature on the non-genetic factors associated with MS prevalence and progression. Moreover, the findings of this study support a link between diet’s overall inflammatory potential and the odds of MS. Furthermore, the findings are in line with the use of DII as a predictor of MS disease. Such promising findings highlight the critical importance of educating MS patients and raising their awareness about food group choices and eating habits in order to improve their health and slow the progression of MS symptoms.

Despite its strengths, this study has some limitations. These includes variations in the duration of illness in MS cases, for which we used simple random sampling. We did limit recruitment only to prevalent cases and disease duration varied from 1 to 3 years, which might affect individuals’ appetites and dietary requirements. Furthermore, the results of this study are limited to a large population-based prevalent case-control study in Jordan. Therefore, questions remain regarding the ability to generalize findings to the MS patient population in Arab countries. Hence, a larger MS patient population and MS subtype shall be included in the analysis from all Arab countries. Additionally, this prevalent case-control study is subject to reporting biases that result from individuals’ knowledge of disease and the effect of disease on factors that, in turn, might affect diet. In addition, the FFQ used in this study could not capture all DII components; hence, more comprehensive FFQ is required.

Although healthy controls without MS matched on sex and age were recruited from the general population, differences were observed with respect to smoking and obesity. This could be a limitation of this population-based prevalent case-control study design. Though we controlled a variety of covariates including age, sex, body mass index, and smoking status, residual confounding still may exist. Moreover, physical activity is an important confounder and needs further future study.

## 5. Conclusion

The major finding of this study is the use of DII as a predictor of MS odds, this could highlight the critical importance of educating MS patients and raising their awareness about food group choices and eating habits in order to improve their health and slow the progression of MS symptoms. Furthermore, DII is an important tool that contribute to overall diet-related inflammation for MS patients with an illness duration, which varied in the previous 3 years. Moreover, this study is consistent with an association between the overall inflammatory potential of diet and odds of having prevalent MS.

## Data availability statement

The original contributions presented in this study are included in the article/supplementary material, further inquiries can be directed to the corresponding author.

## Ethics statement

The studies involving human participants were reviewed and approved by the Research Ethics Committee, University of Petra, Jordan (UOP/REC: Q1R/11/2020) granted ethical approval for the study. The patients/participants provided their written informed consent to participate in this study.

## Author contributions

OA, NLB, and HJ: conceptualization, methodology, writing—original draft preparation, and writing—review and editing. HJ: software and formal analysis. NS and JH: investigation. AY and KT: data curation. OA, HJ, JH, NLB, and KT: funding acquisition. All authors contributed to the article and approved the submitted version.
